# Therapeutic Strategies of Kidney Transplant Ischemia Reperfusion Injury: Insight From Mouse Models

**Published:** 2019-02-20

**Authors:** Longhui Qiu, Zheng Jenny Zhang

**Affiliations:** 1 Comprehensive Transplant Center, USA; 2 Department of Surgery, USA

**Keywords:** Therapies, Kidney, Transplantation, Ischemia Reperfusion Injury, Mouse Models, Cold Ischemic Time, Complement, Regeneration

## Abstract

Ischemia/reperfusion injury (IRI) is inherent to all transplanted organs and is adversely associated with early renal graft function and graft longevity. Despite the progress in immunosuppressive regimens and perioperative care, no FDA-approved treatment for kidney transplant IRI is available to date. In recent years, by utilizing the modified and clinically-relevant mouse models of kidney transplantation (KT_x_) in which extended IRI is induced by the prolonged warm or cold ischemic time, studies have identified several potential therapeutic approaches for KT_x_ IRI, including the hormone supplement, promoting tubular repair and regeneration, and targeting complement system, inflammation, and necroptosis. This review describes some of the lessons learned from mouse models of KT_x_ with regard to factors that influence the severity of transplant IRI and the potential therapeutic targets.

## Introduction

End-stage renal disease (ESRD) is a global public health problem with generally poor outcomes and high costs. According to the latest U.S. Renal Data System Annual Data Report (2018), more than 720,000 Americans are being treated for ESRD. Kidney transplantation (KT_x_) has become the optimal treatment for ESRD. However, all transplanted kidneys inevitably experience ischemia/ reperfusion injury (IRI) - the restriction of blood flow and the restoration of blood supply during transplant process trigger a cascade of noxious events, including the microvascular alteration, reactive oxygen species (ROS) responses, cytokine and chemokine release, and leukocyte activation, resulting in sterile inflammation and acute tubular necrosis (ATN) in renal grafts [1,2]. Severe transplant IRI frequently translates into delayed graft function (DGF), a common complication associated with a high morbidity and mortality post-KT_x_ and allograft rejection [1,3–5].

With a growing gap between kidney demand and supply, the quality of the donor kidney pool continues to deteriorate as the increasing donor age and associated comorbidities accumulate [6,7]. Expanded criteria donor kidneys of perceived lower quality are particularly susceptible to IRI, which compromises graft outcomes [7]. Therefore, seeking strategies to understand the pathophysiology of KT_x_ IRI and to develop effective treatments is highly desirable for transplant recipients [8]. In recent years, our laboratory and others have been attempting to adapt the mouse KT_x_ into a more clinically feasible model to investigate the mechanisms and new therapeutic approaches of transplant IRI. In this mini-review, we discuss some of the lessons learned from mouse models of KT_x_ with regard to factors that influence the severity of transplant IRI and the potential therapeutic targets.

## Clinically Relevant Mouse Models of Kidney Transplantation With Extended IRI

Mouse models of vascularized kidney transplantation have been widely used to dissect mechanism of IRI and transplant rejection. Similarly to the clinic, prolonged cold ischemic time (CIT) induce extended IRI in mouse renal grafts, thus introducing a clinically-relevant model for transplant IRI studies (Table 1) [9–17]. While the warm ischemic time (WIT) in most studies was limited to 30 mins, the CIT ranged from 20 mins to 8 hrs. In some studies, both of the native kidneys of recipient mice were removed during the transplant surgery, while in other studies one of the recipient’s native kidneys was kept in situ until day 4–5 post-transplant. The advantage of removing both native kidneys right away is that the immediate renal graft function will be accessible; however, it may also cause more mortalities during the early post-transplant phases due to the intensive IRI. Concerning the donor and recipient strains, both syngeneic and allogeneic mouse models have been employed for testing IRI treatments. However, since the contributions remain unclear with respect to the all immunity versus donor/recipient genetics in graft IRI, the influence of mouse genetic background should be also taken into consideration when designing studies.

## Hormonal Influence

Sex disparities in kidney IRI tolerance have been proven in both animal systems and human studies [9,18]. By utilizing a mouse KT_x_ model with 8hr CIT, Aufhauser et al. [9] showed that female recipient mice had improved renal ischemia tolerance compared to male recipient mice, which correlated with better transplant outcomes. They found that renal IRI was exacerbated in female mice with estrogen receptor α deficiency, while female mice receiving supplemental estrogen prior to ischemia were protected [9]. Moreover, Gueler et al. [10] showed that the EA-230, an oligopeptide derived from β-human chorionic gonadotropin (β-hCG) lysates, ameliorated renal ischemic injury, improved renal allograft function, and prolonged survival in a mouse model of KT_x_ with 1hr CIT. These studies suggest the potential benefits of hormone supplements in kidney transplant IRI [10].

## Complement System

Several experimental models of acute kidney injury (AKI) induced by clamping have demonstrated a clear-cut role for complement system in renal IRI [19,20] yet only a few studies have tested the effect of complement inhibitors in kidney transplant IRI models [12,21]. Using syngeneic mouse KT_x_ models with 4hr CIT, Zheng et al. showed that the administration of siRNA cocktail solution targeting complement 3, RelB, and Fas significantly reduced the expression of proinflammatory cytokines, interleukin-6 (IL-6), and tumor necrosis factor-α (TNF-α), decreased cell apoptosis, and improved renal function [11]. Recently, Casiraghi et al. [12] showed that BALB/c kidneys transplanted into complement factor b-deficient B6 recipients exhibited reduced IRI and diminished T cell-mediated rejection. The administration of anti-complement factor B antibody to recipients in early post-transplant phases ameliorated both IRI and early adaptive immune responses [12].

## Tubular Repair and Regeneration

It has been accepted that proximal tubule regeneration post-AKI occurs from intrinsic tubule cells [22]. Therefore, seeking strategies to promote tubular repair and regeneration holds promise for ameliorating renal graft damage. Our collaborative studies with Isenberg’s Lab showed that the treatment of renal tubular epithelial cells with a CD47 blocking antibody or CD47-targeting siRNA increased expression of self-renewal transcription factors and promoted cell proliferation [13]. Further studies in mouse models of KT_x_ with 4hr CIT indicated that the treatment with a CD47 blocking antibody increased self-renewal transcription factor expression, decreased tissue damage, and improved renal function compared to that in control mice [13].

## Targeting Innate Immunity, Necroptosis, and Cell Stress Pathways

It is known that IRI triggers a vast array of inflammatory mediators that activate innate immune responses. Toll-like receptors (TLRs) are critical molecules involved in inflammation. By using a mouse KT_x_ models with 1hr CIT, Farrar et al. showed that inhibition of TLR2 with a therapeutic agent (OPN301) significantly decreased acute tubular necrosis and improved renal graft function compared to controls [14]. Necroptosis, a term for programmed cell necrosis, is an emerging entity that might be involved in transplant IRI. This process is characterized by a pathway dependent on the receptor-interacting protein kinase 1 (RIPK1)-RIPK3 complex [23]. A recent study by Lau et al. showed that BALB/c mice receiving RIPK3−/−kidneys had improved renal function and longer survival compared to controls, suggesting that inhibition of necroptosis in donor organs may provide a clinical benefit [15].

Other therapeutic targets testing in the mouse KT_x_ models with prolonged CIT included the nitric oxide-delivering high-density lipoprotein-like nanoparticles (NO-HDL-NPs) and Bβ(15–42), a breakdown product of fibrin that could inhibit leukocyte-endothelial adhesion, as summarized in Table 1 and [16, 17]. Besides the therapeutic approaches summarized above, other potential targets that can be tested in the modified mouse models of KT_x_ include the mitochondrial dysfunction [24] endoplasmic reticulum stress [25] and lipid peroxidation [26]. With regard to the mouse model of KT_x_ IRI, the major limitation is that the surgical procedure is technically challenging and requires extensive microsurgical training. Few people in the world are able to perform this procedure, making it less convenient for translational research. Nevertheless, this model is very attractive as it is powerful and clinically-relevant for the mechanistic investigations and for testing new strategies, thereby offering a great opportunity for identifying new therapies for transplant IRI. Furthermore, compared to mouse AKI models in which no transplant procedure is included, mouse KT_x_ IRI model can efficiently test the mechanisms of kidney intrinsic factors (donor) versus extrinsic factors (recipient).

## Conclusion

The progress of therapies in KTx IRI relies on a better understanding the pathophysiology of renal injury and repair. Mouse models of KTx combined with extended IRI could serve as a powerful tool for exploring the mechanisms of renal graft injury and for testing new therapies for transplant recipients.

## Figures and Tables

**Table 1:** Potential therapies or targets of kidney transplant IRI identified by utilizing the modified mouse models of KT with extended IRI.

Author	Year	Mouse KT_x_ Models	Therapies/Targets	Reference
Hormone				
David et al.	2016	Syngeneic; 8hr CIT; 30 min WIT *	Estrogen	[9]
Gueler et al.	2015	Allogeneic; 1hr CIT; 30 min WIT*	EA-230	[10]
**Targeting Complement System**				
Zhang et al.	2016	Syngeneic; 4hr CIT# †	Complement 3, RelB, and Fas	[11]
Casuraghi et al.	2017	Allogeneic; 20min CIT; 20 min WIT#	Complement factor b	[12]
**Tubular Repair and Regeneration**				
Zhang et al.	2016	Syngeneic; 4hr CIT; 30 min WIT#	CD47	[13]
**Targeting Innate Immunity, Necroptosis and Cell Stress Pathways**				
Farraar et al.	2012	Syngeneic; 30min CIT* †	TLR2	[14]
LAU et al.	2013	Allogeneic; 35–40min (WIT+CIT)#	RIPK3	[15]
Rink et al.	2018	Syngeneic; 4hr CIT; 30 min WIT#	NO-HDL-NPs	[16]
Sörensen et al.	2011	Allogeneic; 45min CIT* †	Bβ(15–42)	[17]

Note:

# Both native kidneys of recipient mice were removed during transplant surgery.

* One native kidney of recipient mice was kept in situ to 4–5 days post-transplant.

† Warm ischemic time was not reported.

CIT: cold ischemic time; WIT: warm ischemic time; TLR: Toll-like receptor; RIPK: receptor-interacting protein kinase; NO-HDL-NPs: nitric oxide-delivering high-density lipoprotein-like nanoparticles.

## Abbreviations:

ESRD: End Stage Renal Disease
KTx: Kidney Transplantation
IRI: Ischemia-Reperfusion Injury
ROS: Reactive Oxygen Species
ATN: Acute Tubular Necrosis
DGF: Delayed Graft Function
CIT: Cold Ischemic Time
WIT: Warm Ischemic Time
β-hCG: β -Human Chorionic Gonadotropin
AKI: Acute Kidney Injury
IL-6: interleukin-6
TNF-α: Tumor Necrosis Factor-α
TLRs: Toll like Receptors
RIPK: Receptor Interacting Protein Kinase
NO-HDL-NPs: Nitric Oxide Delivering High Density Lipoprotein Like Nanoparticles
